# Neurobiological Changes Induced by Mindfulness and Meditation: A Systematic Review

**DOI:** 10.3390/biomedicines12112613

**Published:** 2024-11-15

**Authors:** Andrea Calderone, Desirée Latella, Federica Impellizzeri, Paolo de Pasquale, Fausto Famà, Angelo Quartarone, Rocco Salvatore Calabrò

**Affiliations:** 1 Department of Clinical and Experimental Medicine, University of Messina, Piazza Pugliatti, 98122 Messina, Italy; andrea.calderone95@gmail.com; 2 IRCCS Centro Neurolesi Bonino-Pulejo, S.S. 113 Via Palermo, C.da Casazza, 98124 Messina, Italy; federica.impellizzeri@irccsme.it (F.I.); paolo.depasquale@irccsme.it (P.d.P.); angelo.quartarone@irccsme.it (A.Q.); roccos.calabro@irccsme.it (R.S.C.); 3 Department of Biomedical and Dental Sciences and Morphofunctional Imaging, University Hospital “G. Martino”—Via Consolare Valeria1, 98125 Messina, Italy; ffama@unime.it

**Keywords:** mindfulness, neurobiological correlates, meditation, neurorehabilitation

## Abstract

***Background and Objectives:*** Meditation and mindfulness, rooted in ancient traditions, enhance mental well-being by cultivating awareness and emotional control. It has been shown to induce neuroplasticity, increase cortical thickness, reduce amygdala reactivity, and improve brain connectivity and neurotransmitter levels, leading to improved emotional regulation, cognitive function, and stress resilience. This systematic review will synthesize research on neurobiological changes associated with mindfulness and meditation practices. ***Materials and Methods:*** Studies were identified from an online search of PubMed, Web of Science, Cochrane Library, and Embase databases without any search time range. This review has been registered on Open OSF (n) GV2JY. ***Results:*** Mindfulness-Based Stress Reduction (MBSR) enhances brain regions related to emotional processing and sensory perception, improves psychological outcomes like anxiety and depression, and exhibits unique mechanisms of pain reduction compared to placebo. ***Conclusions:*** This review highlights that mindfulness, particularly through MBSR, improves emotional regulation and brain structure, reduces anxiety, and enhances stress resilience. Future research should focus on diverse populations and naturalistic settings to better understand and optimize these benefits.

## 1. Introduction

Meditation is a mental practice aimed at helping each person concentrate his mind and achieve a deep state of relaxation, clear mental conditions, and inner peace. Meditation is rooted deep in ancient spiritual and philosophical traditions, and has a rich history of propagation spanning thousands of years across various cultures [1]. First adapted in religious contexts by Buddhist, Hindu, and Taoist traditions, meditation eventually broke free from these spiritual roots to become a very familiar component of contemporary mental health and personal development [2]. At the core of meditation lies the cultivation of a higher state of awareness and control over one’s thoughts and emotions. It trains the mind to focus on one object, thought, or sensation, which is important for developing self-regulation and building resilience [3]. One can practice watching thoughts and feelings as they come without being attached to or reacting to them. This creates detachment and calm [4]. Such meditation practiced regularly is likely to increase one’s mental well-being by way of reduction in stress, anxiety, and depression. The non-judgmental awareness of internal experiences brings about a balanced state of mind and, thus, better mental health. One is bound to gain emotional stability and develop poises to deal with difficult situations in life [5]. Other than the mental healing benefits, meditation is also practiced for personal growth, self-realization, and spiritual development. Through this, one can deeply understand themselves and their places in the world. Therefore, it can help start a journey toward self-actualization and self-fulfillment [6]. Several practitioners have experienced deeper insights into things and the feeling of connecting to a larger, all-encompassing universal consciousness through meditation [7]. Several meditation techniques can be practiced based on personal preferences and goals: mindfulness meditation, focusing on current moment awareness, and transcendental meditation involving mantras to ease deep relaxation. Other forms include Zen meditation, which requires correct body posture and breathing; loving-kindness meditation cultivates compassion and equanimity toward oneself and others [8]. Each technique offers unique pathways to mental and emotional well-being, and understanding these methods can help individuals choose the practice that best aligns with their personal needs and aspirations [9]. Among the numerous meditation techniques available that best suit various personal needs and aims, one practice that has gained much attention is mindfulness meditation. This mental practice emphasizes that one focuses his or her attention on the present moment, which features an open and non-judgmental attitude. This practice of mindfulness originated in the ancient traditions of Buddhism through its roots in contemplation, but today it has spread to wide recognition and application in modern psychological and medical practice [10]. In general, mindfulness encourages individuals to witness thoughts, emotions, and physical sensations without becoming entangled in them [11]. This means the act of allowing whatever arises in the present moment to come in without judgment or alteration [12]. This is achieved through meditation processes, where most practitioners sit in silence, focus on breathing or any other point of focus, and every time the mind begins wandering, it is brought back gently [13].

### 1.1. Mindfulness and Its Impact on Mental and Physical Health

**Figure 1:**
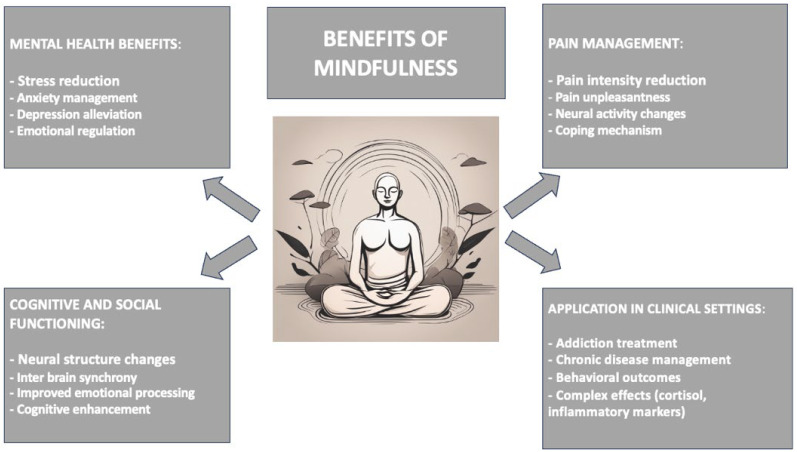
The benefits of mindfulness across different pathologies.

Mindfulness has been empirically shown to have deep impacts on both mental and physical health [14]. It reduces stress, anxiety, and depression, but improves well-being and quality of life [15]. It has been shown that regular mindfulness practice induces structural changes at the neural level, specifically in cortical thickness in areas related to emotional regulation and sensory processing. They are usually accompanied by increased functioning of the mind, which includes reduced anxiety, worry, and depression [16]. Mindfulness also aids in coping with pain. Studies have shown that mindfulness meditation is significantly superior to placebo treatments in reducing both the intensity and unpleasantness of pain [17]. This effect is believed to be derived from the potential of mindfulness to change activity within the brain in areas important for pain perception and emotional processing around the orbitofrontal cortex and anterior cingulate cortex (ACC) [18]. Not only will some research into mindfulness likely improve psychological and physiological benefits, but it is also likely to improve social interaction [19,20]. In particular, advanced brain imaging techniques have shown that mindfulness practitioners increase inter-brain synchrony during face-to-face interactions. This synchrony is evident at particular brain wave frequencies and may indicate a high degree of mutual understanding and connection between people interacting [21]. Deng et al. (2024), in this regard, found that mindfulness meditation produces strong synchrony in the brains of adolescents, particularly when they are experiencing multiple emotional states at the same time. The study observed, through cutting-edge neuroimaging, that meditation increased synchrony in brain regions typically involved in emotional processing and empathy. This increased synchrony is related to better emotional regulation and stronger interpersonal bonding [22]. It has also been applied in a variety of contexts, including addiction treatment and chronic disease management. For example, mindfulness-based interventions have been found to help reduce cigarette cravings and consumption in smokers, particularly in women [23,24]. In the context of chronic diseases, such as multiple sclerosis, mindfulness practices have been linked to improvements in behavioral outcomes and decreases in inflammatory markers; however, most studies have failed to find significant changes in physiological measures, such as cortisol [25,26,27]. While this has helped to show a reduction in impulsivity and some neural correlates, its effects on these variables are indeed complex and sometimes even conflicting [28,29]. Long-term meditators may differ from non-meditators in terms of impulsivity and brain connectivity; however, these findings are not always replicable across studies [30]. A synthesis of the benefits of mindfulness across different pathologies is presented in Figure 1. In its MBSR format, mindfulness has developed over the years into perhaps the best-known and most empirically supported approach to integrating mindfulness practices into healthcare and therapeutic settings. MBSR is an evidence-based, structured program for the cultivation of mindfulness through meditation practices and exercises that aim to develop body awareness. It was developed in 1979 at the University of Massachusetts Medical School by Dr. Jon Kabat-Zinn. From its conceptualization to help chronically pain-inflicted patients and those with stress disorders, the breadth of physical and mental health conditions it currently addresses makes the program one of the most popular and researched mindfulness interventions in the world [31,32]. The MBSR program typically runs for eight weeks. For this duration, participants meet once a week with the group; this group session is about 2.5 h long. Guided mindfulness meditation, some easy yoga exercises, and body scan exercises are held within the sessions; one allows time to be directed toward breathing, sensations in the body, thoughts, and emotions without judgment [33,34]. Supplementing these sessions, participants are asked to commit to daily mindfulness practice at home, but the recommended duration is approximately 45 min of formal meditation per day [35,36]. One of the central components of MBSR is the cultivation of a mindful attitude characterized by openness, curiosity, and acceptance of the present moment—whatever the experience is—whether pleasant, unpleasant, or neutral. Thus, the attitude adopted places one in a better position to know one’s automatic responses to difficulties and stressors and to deal with them skillfully, without falling into impulsive reactions. It has been proven that MBSR is effective in reducing stress symptoms, anxiety, and depression [37,38]. This program has also been proven efficient in assuaging chronic pain [39,40]. Many research studies have shown that participants often divulge greatly improved psychological well-being, like reduced ruminations, enhanced emotional regulation, and increased resilience [41,42]. In addition, MBSR has been associated with positive changes in brain structure and function levels, especially regarding attention, self-mention, and emotion regulation. It has also been tailored for different populations, including healthcare professionals and students, as well as those in highly stressed professions [43]. Clinical situations also use it to augment the conventional treatment of cancer, cardiovascular diseases, and autoimmune disorders [44].

### 1.2. Mindfulness and Neuroplasticity: Structural Changes in the Brain

The mindfulness practice fields have been well researched, especially in the last couple of years, regarding their neurobiological effects. Quite several changes in structure and function were identified in the brain. The consistent practice of mindfulness meditation results in neuroplasticity, which brings about observable modifications in different areas of the brain, associated with managing emotions, focusing, and being conscious of oneself. It has been demonstrated that increasing the production of brain-derived neurotrophic factor (BDNF) can support neuroplasticity. A higher amount of BDNF leads to a longer lifespan, growth of neurons, and synaptic plasticity, enhancing learning and memory. One of the best revelations that has been well-documented is the increased cortical thickness, mainly in the prefrontal cortex and anterior cingulate cortex (ACC) [45,46]. Where information on executive functions, such as decision-making and problem-solving, is stored, the prefrontal cortex is involved; moreover, the ACC is involved in critical aspects of attention and self-regulation [47]. Such structural changes might indicate that mindfulness strengthens the parts of the brain essential for maintaining control over the physiology of stress and emotional responses [48]. Another important discovery regards the changes in the amygdala, a region associated with the processing of emotions, in particular fear and stress. Specifically, it has been determined that mindfulness can lead to a reduction in size and reactivity in the amygdala, which is in line with reports of reduced levels of stress and anxiety [49,50]. This downregulation of the amygdala is also associated with an improved capacity for the regulation of affective responses, leading to a calmer and more resilient mind [51]. Mindfulness also appears to connect more robustly and functionally across different brain regions. Functional magnetic resonance imaging (fMRI) studies have shown increased connectivity between the prefrontal cortex and the default mode network (DMN), which is a network of brain areas in self-referential thoughts and mind-wandering [52,53]. In meditators, activity in the DMN is lowered during meditation, which has been connected to a lesser degree of ruminations and mind-wandering, typical contributors to anxiety and depression [54,55].

### 1.3. Mindfulness and Neurochemical Balance: GABA, Serotonin, and BDNF

**Figure 2:**
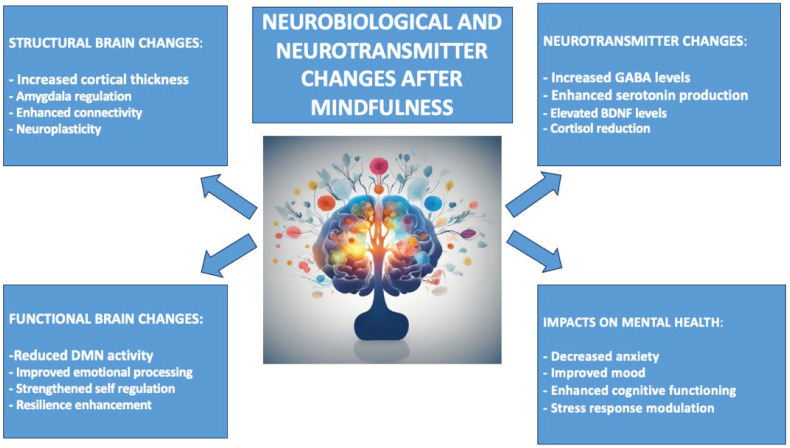
The neurobiological and neurotransmitter changes following mindfulness.

Mindfulness techniques affect neurotransmitter systems in the brain. For example, individuals who regularly practice meditation have been found to have higher levels of GABA. Since the neurotransmitter GABA works to minimize neural activity, more significant levels of this chemical will help reduce anxiety in an individual while at the same time improving his or her mood [56,57]. Evidence of higher levels of serotonin production has also been associated with mindfulness. Serotonin functions in the body to aid in regulating a person’s mood and general well-being feelings [58,59]. On the other side, higher levels of BDNF have been related to increased cognitive functioning and emotional resilience [60,61,62]. Lastly, the practice of mindfulness is associated with lower cortisol levels, the major hormone related to stress, a finding that is already by itself enough to recommend a decrease in bodily stress response [63,64,65]. This reduction in cortisol, coupled with the above-mentioned neurobiological alterations, brings out the deep impact of mindfulness on the brain and the body, thereby impacting total mental health and well-being. These findings all point to one thing: that mindfulness does not only improve psychological health but also changes the brain in general and in a long-lasting way, supporting better emotional regulation, cognitive function, and resilience against stress [66,67,68]. A summary of neurobiological and neurotransmitter changes due to mindfulness is shown in Figure 2.

### 1.4. Mindfulness Techniques and Their Applications

Apart from mindfulness meditation, there are various techniques in the field of meditation that provide distinct benefits and specific methods for improving mental health. For example, Transcendental Meditation (TM) utilizes particular mantras to induce deep relaxation and increased awareness, enabling individuals to surpass their usual thinking patterns and encounter deep states of peace and tranquility. This method has been proven to greatly decrease stress and enhance overall quality of life, which is why it is a favored option for individuals seeking relief from everyday stressors. Zen meditation (Zazen) is a different method that focuses on the importance of proper posture and controlling the breath. Practitioners usually sit quietly, concentrate on their breath, and develop a non-judgmental awareness of their thoughts as they come up. This type of meditation improves focus and promotes being present, which helps emotional control. Practicing Metta meditation fosters compassion and benevolence toward oneself and others. By engaging in specific phrases and mental images, individuals develop a mindset of love and compassion, which leads to higher levels of positive feelings and better connections with others. Body Scan Meditation aims to increase mindfulness of physical sensations, encourage calmness, and strengthen the mind-body relationship. This method is especially helpful for people dealing with persistent pain, as it enhances awareness of bodily sensations and promotes the acceptance of physical sensations. Through the comprehension of these diverse methods, people can enhance their ability to choose a meditation routine that suits their individual tastes and objectives. Every approach provides different ways to improve mental and emotional health, underscoring the significance of identifying the most suitable option for individual requirements and goals.

This systematic review will synthesize research on the neurobiological changes associated with mindfulness and meditation practices.

## 2. Materials and Methods

### 2.1. Search Strategy

**Figure 3:**
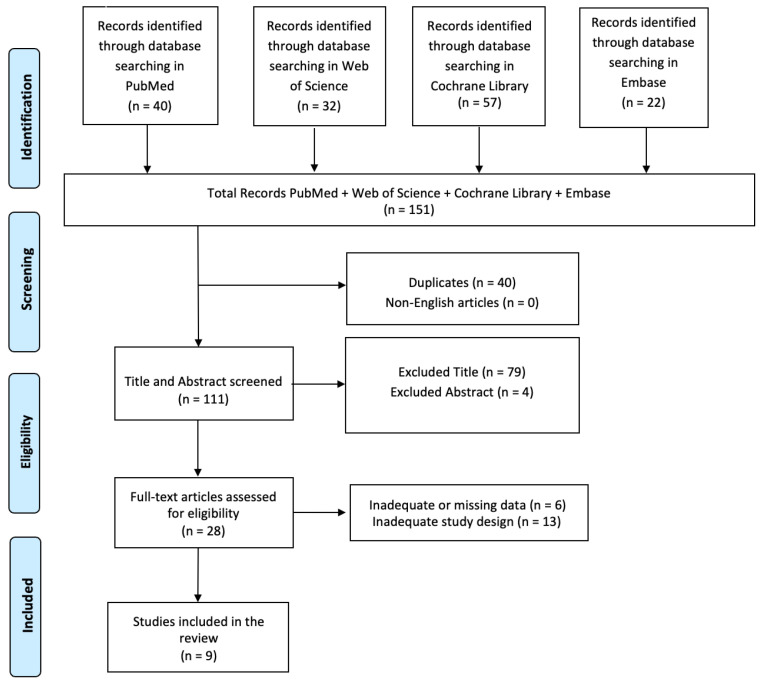
PRISMA 2020 flow diagram of evaluated studies.

A comprehensive literature search was performed using PubMed, Web of Science, Cochrane Library, and Embase databases, employing the keywords (All Fields: “Mindfulness”) AND (All Fields: “Neurobiological correlates”), without any specific search time range. The PRISMA (Preferred Reporting Items for Systematic Reviews and Meta-Analyses) flow diagram was utilized to outline the process (identification, screening, eligibility, and inclusion) for selecting relevant studies, as illustrated in Figure 3. The titles and abstracts of the database searches were independently reviewed. Articles were evaluated for their relevance based on predefined inclusion criteria. Titles and abstracts that met these criteria were fully reviewed. Multiple expert teams independently selected articles, analyzed the data to minimize bias, and discussed discrepancies until a consensus was reached. This review has been registered on Open OSF (n) GV2JY.

### 2.2. PICO Evaluation

We applied the PICO (Population, Intervention, Comparison, Outcome) model to create the search terms. The population included people participating in different mindfulness and meditation practices, including MBSR, performed across both clinical and nonclinical populations. Neurobiological outcomes will be measured based on neuroimaging techniques such as fMRI and magnetic resonance imaging (MRI), electrophysiological techniques such as electroencephalogram (EEG), and other biological markers like neurotransmitter levels and cortical thickness. The comparison would consist of individuals not engaging in mindfulness, those receiving standard care, or alternative therapeutic interventions. The outcome includes neurobiological changes associated with mindfulness measured through neuroimaging techniques, electrophysiological assessments, and other biological markers. Key outcomes include changes in brain structure and function, neuroplasticity, and alterations in stress-related biomarkers.

### 2.3. Inclusion Criteria

A study was included if it described or examined the neurobiological changes associated with mindfulness and meditation practices. Only articles written in English were included in this study. Additionally, studies that described or investigated the functional assessments of these patients were included. We only included studies conducted in human populations and published in English that met the following criteria: (i) original or protocol studies of any kind, and (ii) articles that detail the neurobiological changes associated with mindfulness and meditation practices.

### 2.4. Exclusion Criteria

A study was excluded if it lacked data or information regarding the neurobiological changes associated with mindfulness and meditation practices. Systematic, integrated, or narrative reviews were also excluded; however, their reference lists were reviewed and included when relevant. Additionally, any articles written in languages other than English were excluded.

## 3. Results

In total, 151 articles were found: 40 articles were removed due to duplication after screening, 0 articles were excluded because they were not published in English, and 83 articles were excluded based on title and abstract screening. Finally, 19 articles were removed after screening for inadequate and untraceable study designs (Figure 3). Nine research articles met the inclusion criteria and were therefore included in the review. The studies are summarized in Table 1. The studies discussed in this review analyzed the neurobiological changes associated with mindfulness and meditation practices. Nine articles explored the diverse impacts of mindfulness practices on brain activity [69,70,71,72,73,74,75,76,77].

### 3.1. The Diverse Impact of Mindfulness Practices on Brain Activity

Researchers have investigated the effects of MBSR on neural and psychological outcomes, a rather broad field with a focus on changes in brain structure, emotional regulation, and pain perception. These studies have been conducted using randomized controlled trials and observational designs and have provided insights into how MBSR enhances the brain areas generally responsible for emotional processing, sensory perception, and cognitive control. In the first randomized clinical trial, neuroanatomical changes and associated changes in psychological variables related to an 8-week MBSR course were compared between 23 meditation-naïve individuals and a waiting list control group of comparable age and sex. The cortical thickness of the right insula and somatosensory cortex was significantly increased in the MBSR trainees. State anxiety, worry, depression, and alexithymia paralleled these increases with a significant decrease. This was associated with greater thickness of the right insula and decreased alexithymia [69]. In one study, 75 healthy volunteers were randomized to one of four interventions: mindfulness meditation versus placebo conditioning versus sham mindfulness meditation versus book-listening control. The current study found that all cognitive manipulations significantly reduced pain intensity and unpleasantness relative to the control group. In particular, mindfulness meditation decreased both pain intensity and unpleasantness significantly more than placebo analgesia or sham mindfulness meditation. On fMRI, the mindfulness meditation group showed increased activity in brain regions of a priori interest associated with the cognitive modulation of pain, specifically in the orbitofrontal and anterior cingulate cortices. In contrast, the neural mechanisms of placebo analgesia were found in sensory processing regions and the dorsolateral prefrontal cortex. This is the first evidence to date that mindfulness meditation engages a distinctive mechanism of pain reduction, independent of placebo treatment [70].

### 3.2. Neurobiological Outcomes of Mindfulness Interventions

In another randomized controlled trial of 67 smokers, 33 were randomized to the mindfulness training (MT) group and 34 to the active control group. App-delivered MT significantly reduced posterior cingulate cortex reactivity to smoking cues relative to the control. This decrease was related to a decrease in cigarette consumption that was not engendered in the control group for the MT participants. This effect was specifically sex-related; in women, the reduction in posterior cingulate cortex reactivity was related to reduced smoking, whereas no such association was observed in men [71]. In an eight-week mindfulness intervention with 60 participants, mindfulness practice had no significant effects on impulsivity, as operationalized by the go/no-go task and the Barratt Impulsiveness Scale, or on neural correlates of impulsivity (frontostriatal gray matter, functional connectivity, and dopamine levels) relative to active or wait-list controls. In the go/no-go task, long-term meditators did not differ from meditation-naïve participants; however, they showed less attention, and more motor and non-planning impulsivity. Moreover, long-term meditators had less gray matter in the striatum and higher cortico-striatal-thalamic connectivity, together with a lower spontaneous eye-blink rate, than meditation-naïve participants [72]. In an observational study of 23 patients with multiple sclerosis, an MBSR program improved several behavioral outcomes and increased the size of the right hippocampus. This study also showed that greater inflammatory gene expression, indexed by the conserved transcriptional response to adversity profile, was associated with worse patient-reported anxiety, depression, stress, loneliness, and lower eudaimonic well-being. In contrast, hair cortisol did not differ significantly before and after the MBSR program [73]. Braden et al. conducted a nonrandomized trial with a sample of 12 subjects who received a shorter, 4-week MBSR course compared to 11 controls (reading material about reduction of stress) and found significant improvements in overall depression in both groups. Overall, the MBSR group was the only group able to achieve significant reductions in back pain and somatic-affective depression symptoms. The MBSR team also demonstrated significant boosts in frontal lobe blood flow linked to better detection of emotional shifts. Another inference drawn was that the shortened MBSR course was good for pain relief and emotional awareness, but the 8-week traditional MBSR course might be necessary to see wider benefits in anxiety and cognitive aspects of depression [74]. In a study of long-term meditators of Acem meditation, fMRI was used to explore brain activation at rest and during the presentation of a meditation sound. Compared with the concentrative control tasks, it was found that the meditation sound evoked remarkably higher activation of the bilateral areas in the inferior frontal gyrus. This was not a decreasing activation over time; on the contrary, activation increased with the length of the meditation sessions. The findings show that meditation with relaxed focus is associated with parts of unique activation in the prefrontal cortex, distinguishing it from other cognitive tasks, and increasing knowledge about the neurobiological mechanisms underlying meditation [75]. In a group of 140 healthy adults, the researchers examined the association between mindful awareness and brain activity in an extremely localized manner within the prefrontal cortex and subgenual ACC. Brain glucose metabolism can be used to determine how personality influences brain function. These findings reverse the effects of temperament on metabolism in this brain area, which is related to anxiety and vulnerability. In contrast, a personality trait called self-transcendence, which is associated with spirituality and intuitiveness, has the opposite effect on the same area of the brain. Furthermore, an analysis of the whole brain revealed that high spirituality is significantly associated with higher metabolism in that area of the brain [76]. One last article investigated the neural correlates of mindfulness in the context of social interactions; they recorded brain activity using a portable EEG while individuals were engaged in face-to-face conversations as part of an exhibit in a museum. While failing to replicate earlier work associating specific mindfulness with brain responses produced in a laboratory environment, they showed that self-reported mindful awareness was associated with increased inter-brain synchrony between interacting individuals. This synchrony was visible in both theta and beta frequency bands. This study: therefore, underlines the need for the study of mindfulness under more naturalistic social conditions, where although individual brain responses may not agree with previous findings on social traits, the potential of mindfulness in driving inter-brain synchrony when interacting with others is evident. This further cautions the use of commercially available EEG systems in research related to the neural correlates of social traits [77]. In summary, MBRS can enhance brain areas related to emotional processing and sensory perception and improve psychological outcomes like anxiety and depression.

### 3.3. The Complexities of Mindfulness: Emotional Regulation and Impulsivity Outcomes

In addition, MBSR can increase the cortical thickness. Mindfulness has dissimilar neural mechanisms of pain reduction in comparison with placebo treatments for pain reduction. Mindfulness training also helps reduce smoking cue reactivity, particularly in women, and enhances emotional regulation. However, its effect on impulsivity is still unclear, as long-term meditators both provide evidence and fail to provide evidence of its effects on impulsivity and neural connectivity. It is also the case that mindfulness practice gives rise to better emotional awareness and social interaction, as shown by enhanced inter-brain synchrony during naturalistic interactions.

## 4. Discussion

This systematic review investigated neurobiological changes associated with mindfulness and meditation practices. The results highlight the broad benefits of MBSR across neuroanatomical, psychological, and social domains. MBSR programs, including abbreviated versions, have been shown to increase cortical thickness in areas such as the right insula and improve psychological outcomes like reduced anxiety, depression, and alexithymia. Moreover, mindfulness meditation has a specific effect on reducing pain and improving emotional awareness; research indicates that it activates different brain mechanisms than placebo treatments. It also shows promise for smoking cessation, particularly in women, by lowering brain reactivity to smoking cues. While mindfulness does not consistently affect impulsivity, it benefits social interactions by increasing inter-brain synchrony during conversations [69,70,71,72,73,74,75,76,77]. Therapies, such as MBSR, have demonstrated the ability to induce neurobiological changes that could directly benefit neurorehabilitation. First, the increases observed in the cortex, particularly in areas including the right insula and somatosensory cortex, would indicate improved sensory and interoceptive processing. This could be particularly useful during neurorehabilitation, as patients often face sensory deficits or altered body awareness, as in the case of stroke and traumatic brain injury [78,79]. This is especially true given that the finding that mindfulness practice also modulates pain through mechanisms separate from placebo effects speaks to its utility in managing chronic pain conditions that are often comorbid with neurological impairments, including both spinal cord injury and multiple sclerosis [80,81]. Simpson et al. (2023) targeted participants with multiple sclerosis, but in their study, they found that mindfulness-based interventions result in neurobiological changes of relevance for improved quality of life. Functional neuroimaging has revealed increased connectivity in the structures responsible for emotional regulation and brain function, such as the DMN and executive control areas. These changes were associated with reductions in the symptoms of depression and anxiety, thus proving that mindfulness alters brain activity in a manner conducive to improved well-being in multiple sclerosis [82].

### 4.1. Pain, Stress, & Anxiety

Regarding pain, Hunt et al. (2023) showed that mindfulness-based treatments for episodic migraines are accompanied by considerable neurobiological changes. As meditation practice continued, decreases in both the frequency and intensity of migraines were linked to alterations in brain areas known to be involved in pain perception and regulation, such as the ACC and insula. This may mean that mindfulness practices alter neural pathways involved in the processing of pain, thereby contributing to relief from episodic migraines [83]. Since mindfulness practices reduce pain perception through the prefrontal cortex and anterior cingulate cortex, interventions could benefit patients through pain management, as well as through the simultaneous improvement of emotional regulation, which is an important predictor of rehabilitation outcomes, reducing symptoms of anxiety, stress, and depression [84,85,86,87]. Pagni et al. (2023) were found to report that MBSR had a significant effect in reducing symptoms of anxiety in adults with autism. They detected neurophysiological changes at the level of brain wave patterns and increased connectivity in the areas of emotional regulation, by which MBSR might help manage anxiety in autism [88]. Similarly, a broader perspective on anxiety disorders has provided additional neurobiological insights related to mindfulness-based interventions. A systematic review conducted by Gerber and Matuschek (2023) on mindfulness-based interventions concerning anxiety disorders found that such interventions can bring about significant changes in these neuroanatomical stress vulnerabilities, including amygdala and prefrontal cortex activation [89]. Based on these findings, short-term mindfulness interventions have also shown significant neurobiological effects. Diez et al. (2023) identified the benefit of a short-term mindfulness and compassion retreat in improving stress reduction and mental well-being. Their study emphasized the interplay between gene expression pathways activated in stress responses, with an observed increase in neural activity in regions associated with emotional regulation [90]. Bakshi and Srivastava (2024) explained the neurobiological understanding of yoga and mindfulness, and their effects on neuronal function, stress, and well-being. Their review showed that the impact of mindfulness and yoga on the brain areas responsible for regulating stress, emotional control, and cognitive processes is positive. More specifically, function is enhanced in areas like the prefrontal cortex and amygdala, which substantiate emotional resilience and improve cognitive control. They further noted positive effects on brain health in settings related to addictive disorders and palliative care, which means that mindfulness might have a bearing on neural circuits underpinning stress response and reward processing [91]. Siew and Yu (2023) conducted a meta-analysis of structural brain changes discovered in mindfulness-based randomized controlled trials. They reported that mindfulness practice is associated with increased cortical thickness and changes in brain areas responsible for attention and self-regulation [92].

### 4.2. Healthy Lifestyle and Well-Being

In the context of brain health, Mace et al. (2024) conducted a systematic review of the effects of mindfulness-based interventions on lifestyle behaviors. Mindfulness-based interventions, according to their findings, have resulted in neurobiological changes that underlie a healthy lifestyle. More specifically, mindfulness practices are associated with better functioning in self-regulation- and executive function-related areas of the brain, such as the prefrontal cortex. These changes encourage better involvement in health-enhancing behaviors, and thus foster improved brain health [93]. In addition to their effects on lifestyle, mindfulness interventions have been studied for their roles in functional connectivity and neural processes. Hammersjö Fälth and Eklind (2024) conducted a systematic review of the effects of cognitive behavioral therapy and MBSR on functional connectivity. They discovered that although both therapies are known to alter brain connectivity, MBSR is associated with increased connectivity in regions related to self-awareness and parts of the emotional regulation network, while cognitive behavioral therapy alters regions involved in cognitive control and emotional processing [94]. Overall, the results provide evidence for the induction by mindfulness and meditation practice of significant neurobiological changes at several levels of experiential processing, from cortical structure and connectivity to emotional and cognitive processing. Since neurological damage is usually accompanied by psychological trauma, one of the positive roles of mindfulness interventions in neurorehabilitation may be related to improved mental health, which can help increase patient motivation and engagement [95]. Such mindfulness practice can improve emotional resilience during recovery by reducing anxiety, depression, and alexithymia, thus playing an important role in the long-term rehabilitation process [96,97]. Finally, mindfulness was found to enhance functional connectivity in a network of interconnected regions that covered cognitive control and self-regulation [98]. In particular, this may be an important application in cognitive rehabilitation, where executive function and attentional control must be regained [99,100]. In this respect, improved connectivity within networks, such as the default mode network and regions of emotional regulation, may allow for the restoration of cognitive and behavioral functioning in patients [101]. In summary, neurorehabilitation, in combination with mindfulness, will result in improved sensory processing, manageable pain, emotional regulation, and cognitive control, all factors that aim to ensure better recovery and improvement in the quality of life of persons with neurological impairment [102,103].

### 4.3. Neurobiological Benefits of Mindfulness Alternatives in Neurorehabilitation

Other options besides MBSR involve practices like yoga, diaphragmatic breathing, biofeedback, guided imagery, Tai Chi, and Qigong, which influence brain function and structure. These approaches, while varied, encompass common elements related to mindfulness concepts, such as self-awareness, self-regulation, and mind-body integration, which have shown the ability to promote neuroplastic alterations. Understanding these neurobiological effects is essential to acknowledge the benefits of neurorehabilitation. Yoga, for instance, includes a blend of physical poses (asanas), breathing exercises (pranayama), and meditation, which activate the brain’s sensory and motor pathways alongside regions associated with emotional control, such as the prefrontal cortex and limbic system. Neuroimaging research indicates that yoga activities enhance connectivity between the prefrontal cortex and limbic areas, which is crucial for managing stress and emotions [104,105,106]. Diaphragmatic breathing influences the autonomic nervous system, especially the parasympathetic part, by activating the vagus nerve. This activity boosts heart rate variability, which is associated with better emotional control and decreased physiological stress reactions. The soothing influence on the autonomic nervous system assists in reducing the sympathetic “fight or flight” reaction, encouraging a feeling of relaxation and mental clarity. In neurorehabilitation, diaphragmatic breathing may help to reduce anxiety and boost concentration, which in turn enhances patient participation in therapy [107,108]. Biofeedback, involving the immediate tracking of physiological responses, such as heart rate, muscle tension, or skin conductivity, instructs patients on how to consciously control these reactions using different methods, including breathing exercises or muscle relaxation techniques. This type of self-regulation may result in enduring alterations in brain function by enhancing the link between the brain and body, especially in regions associated with emotional regulation and executive control. Studies have indicated that biofeedback can increase activity in the prefrontal cortex, an area involved in decision-making, emotional control, and advanced cognitive function [109,110]. Guided visualization is an additional method that, while not explicitly mindfulness, aligns with its fundamental principles. By mentally practicing tranquil images or imagining preferred results, individuals can stimulate brain regions linked to emotion control and sensory processing, including the amygdala and the occipital cortex. Visualization has been demonstrated to encourage neural alterations similar to those seen in mindfulness practice, creating a calm state that can enhance pain control and reduce anxiety [111,112]. Tai Chi and Qigong, traditional practices from China that integrate movement, breath management, and meditation, have been discovered to affect brain regions linked to motor control, balance, and emotional regulation. Tai Chi has demonstrated the ability to enhance motor coordination and balance, which are crucial for individuals healing from neurological issues such as stroke or Parkinson’s disease. Neuroimaging research has shown that Tai Chi enhances brain activation in regions like the basal ganglia, which govern motor control, and the prefrontal cortex, which is associated with executive function and focus [113,114,115]. To summarize, yoga, diaphragmatic breathing, biofeedback, guided visualization, Tai Chi, and Qigong all encompass neurobiological processes that promote self-regulation, emotional strength, and neuroplasticity.

### 4.4. Strengths & Limitations

This systematic review has several strengths. It focuses on neurobiological changes due to the practice of mindfulness and meditation through a thorough database search, which includes PubMed, Web of Science, Cochrane Library, and Embase. Moreover, it encompasses a broad range of studies and techniques, such as MBSR, and applies rigorous criteria for the studies to be included in the review that cater to specific neurobiological changes through neuroimaging and electrophysiological techniques. This systematic review is only an overview of research on how mindfulness is linked to brain structures and functions, thus leading to better pain management, smoking cessation, and outcomes of chronic diseases. However, reviews of these types have limitations: differences in study designs, populations, and methodologies limit the drawing of integrated results and generalization of conclusions. This could compromise the reliability of the findings, as they rely heavily on small sample sizes in some of the studies and on differences between short-term practices and the long-term effects of the practices of mindfulness. A focus on specific populations and settings has implications for the generalizability of the results. Moreover, disparities related to impulsivity indicate that further research is needed. Finally, limiting the search to publications in English might have excluded relevant research articles on the current topic published in other languages and, as a consequence, limited the review’s comprehensiveness.

## 5. Conclusions

This review discusses the neurobiological changes attributed to mindfulness and meditation practices regarding their impact on mental health and changes in brain structure. It emphasizes increased cortical thickness within emotion regulation and sensory processing areas, reduced level of anxiety, and improved regulation of emotions. Mindfulness also enhances the functioning of the brain through improvement in connectivity and increasing neurotransmitter systems, hence improving mood and reducing anxiety. Future studies should analyze diverse populations, study designs, and the impact of mindfulness on a variety of mental health disorders. Advanced neuroimaging and electrophysiological techniques can be combined to probe the neural mechanisms underlying mindfulness. Moreover, further research on mindfulness effects within naturalistic settings and cultural contexts will greatly improve the applicability and understanding of such practices. While the review recommends that the major benefits of mindfulness on mental and physical health are involved, more studies are needed to completely understand its neurobiological effects and maximize the efficacy of its clinical applications.

## Figures and Tables

**Table 1:** Summary of studies included in the research.

Author	Aim	Study Design	Treatment Period	Sample Size	Outcomes Measures	Main Findings	Statistical Analyses
Santamecchi et al., 2014 [69]	To investigate the neuroanatomical and psychological changes in individuals before and after an 8-week MBSR training program, focusing on both cortical and subcortical brain changes as well as multiple psychological dimensions.	Longitudinal Study.	8 weeks.	23 meditation-naïve subjects.	BDI-II, TAS-20, PSWQ, STAI, MAAS.	The MBSR participants experienced a notable rise in cortical thickness in the right insula and somatosensory cortex. There was also a notable reduction in psychological indices related to worry, state anxiety, depression, and alexithymia. Additionally, a correlation was found between the increase in right insula thickness and the decrease in alexithymia levels.	Multivariate pattern classification approaches were used to identify clusters of brain regions responsive to MBSR training. Correlational analyses were conducted to link neuroanatomical changes with psychological improvements.
Zeidan et al., 2015 [70]	To determine whether the analgesic mechanisms of mindfulness meditation are distinct from those of the placebo effect.	Randomized Controlled Trial.	4 days.	75 healthy human volunteers.	fMRI.	Mindfulness meditation significantly reduced pain intensity and unpleasantness more than placebo analgesia and sham mindfulness meditation. This pain relief was linked to activation in brain regions associated with cognitive pain modulation, such as the orbitofrontal, subgenual anterior cingulate, and anterior insular cortex.	Statistical comparisons of pain intensity and unpleasantness ratings, with significant results indicated by *p*-values less than 0.05. Neural activity associated with each intervention was analyzed through functional neuroimaging.
Janes et al., 2019 [71]	To evaluate whether MT delivered via a smartphone app could reduce PCC reactivity to smoking cues and if changes in PCC reactivity were associated with smoking reduction.	Randomized Controlled Trial.	One Month.	67 participants, with 33 in the MT group and 34 in the control group.	fMRI.	The MT group showed a significant correlation between reduced PCC reactivity to smoking cues and a decrease in cigarette consumption, particularly in women.	Correlational analyses were used to link changes in PCC reactivity with smoking outcomes, with significance levels reported for overall and sex-specific correlations.
Korponay et al., 2019 [72]	To evaluate whether an eight-week mindfulness intervention could reduce impulsivity and affect related cognitive and neural correlates compared to control groups.	Randomized Controlled Trial.	Eight weeks.	105 meditation naive participants and 31 long-term meditators.	BIS-11.	The eight-week mindfulness intervention did not reduce impulsivity or alter neural correlates of impulsivity. Long-term meditators reported lower attentional impulsivity but higher motor and non-planning impulsivity compared to meditation-naïve participants and exhibited different neurobiological profiles.	Analyses compared impulsivity and neurobiological metrics and assessed changes in these metrics following the mindfulness intervention.
Hemond et al., 2024 [73]	To explore the psychological, biological, and neuroarchitecture changes linked to a MBSR program in MS patients.	Prospective Observational Study.	8 weeks.	23 patients with MS.	MRI, BIPS, DASS-21.	The MBSR program was associated with significant improvements in various behavioral outcomes and an enlargement of the right hippocampus head. The CTRA profile revealed that higher inflammatory gene expression correlated with worse anxiety, depression, stress, and loneliness, alongside lower eudaimonic well-being.	Statistical analyses involved comparing pre- and post-MBSR intervention measures, including MRI structural changes, behavioral outcomes, and inflammatory markers as indexed by the CTRA profile.
Braden et al., 2016 [74]	To determine if an abbreviated 4-week MBSR course improves symptoms in chronic back pain patients and to examine the neural and behavioral correlates of MBSR treatment.	Pilot pseudorandomized controlled trial.	4 weeks.	23 participants.	BDI-II.	The MBSR group showed significant improvements in back pain and somatic-affective depression symptoms, along with increased regional frontal lobe hemodynamic activity related to emotional awareness. Both groups saw improvements in total depression symptoms, but the MBSR group had unique benefits in specific areas.	Statistical analyses compared pre- and post-intervention measures within and between groups, assessing changes in self-reported symptoms and fMRI data.
Davanger et al., 2010 [75]	To assess whether brain activation during relaxed focusing on a meditation sound could be distinguished from similar, concentrative control tasks.	Clinical Study.	Not Specificated.	Four advanced male practitioners.	fMRI.	BA47 showed noticeably higher levels of activity when participants repeated a meditation sound in comparison to when they performed concentrative meditation-style cognitive tasks. Additionally, meditation-specific brain activation increased in strength over continuous meditation bouts rather than habituating over time.	The statistical analyses used software for data preprocessing and modeling the expected hemodynamic response with a canonical function.
Hakamata et al., 2013 [76]	To explore the neural basis of personality, particularly examining the interplay between character and temperament and their effects on the sgACC and vmPFC.	Observational Study.	There was no treatment period involved, as the study was cross-sectional in nature, focusing on a single measurement point of brain glucose metabolism and personality traits.	140 healthy adults.	TCI, PET.	HA was negatively correlated with sgACC/vmPFC glucose metabolism, while ST was positively correlated. Individuals with high HA and high ST had sgACC/vmPFC glucose metabolism comparable to those with low scores on both traits.	Region of interest and whole brain analyses were conducted.
Chen et al., 2022 [77]	To investigate the neural correlates of trait mindful awareness during naturalistic social interactions, focusing on how mindfulness affects both individual brain activity and inter-brain synchrony during face-to-face interactions.	Naturalistic observational design.		379 individuals for individual brain response analysis and 62 dyads for the dyadic inter-brain synchrony analysis.	EEG.	The study did not replicate previous laboratory-based findings linking trait mindfulness to individual brain responses. Nevertheless, it was discovered that self-reported mindfulness was linked to higher levels of dyadic inter-brain synchrony in theta and beta frequencies (specifically around 5–8 Hz and 26–27 Hz) during in-person communication.	Pearson correlation coefficients to assess the relationship between mindful awareness scores and inter-brain coupling across different frequency bins.

Legend: Mindfulness-based stress reduction (MBSR), multiple sclerosis (MS), Conserved Transcriptional Response to Adversity (CTRA), Magnetic resonance imaging (MRI), Brief Inventory of Perceived Stress (BIPS), Depression, Anxiety, and Stress Scale (DASS-21), electrocardiographic (ECG), electroencephalographic (EEG), Beck Depression Inventory-II (BDI-II), Toronto Alexithymia Scale (TAS-20), Penn State Worry Questionnaire (PSWQ), State-Trait Anxiety Inventory (STAI), Mindful Attention Awareness Scale (MAAS), Functional Magnetic resonance imaging (fMRI), Bilateral areas of the inferior frontal gyrus (BA47), mindfulness training (MT), posterior cingulate cortex (PCC), Barratt Impulsiveness Scale (BIS-11), subgenual anterior cingulate cortex (sgACC), ventromedial prefrontal cortex (vmPFC), Temperament and Character Inventory (TCI), positron emission tomography (PET), Harm avoidance (HA), self-transcendence (ST).
